# Generation and Characterization of a genetic zebrafish model of SMA carrying the human *SMN2 *gene

**DOI:** 10.1186/1750-1326-6-24

**Published:** 2011-03-28

**Authors:** Le T Hao, Arthur HM Burghes, Christine E Beattie

**Affiliations:** 1 Dept of Neuroscience and Center for Molecular Neurobiology, The Ohio State University, 1060 Carmack Rd., Columbus OH 43210. USA; 2 The Dept of Molecular and Cellular Biochemistry, The Ohio State University, 1645 Neil Av, Columbus OH 43210. USA

## Abstract

**Background:**

Animal models of human diseases are essential as they allow analysis of the disease process at the cellular level and can advance therapeutics by serving as a tool for drug screening and target validation. Here we report the development of a complete genetic model of spinal muscular atrophy (SMA) in the vertebrate zebrafish to complement existing zebrafish, mouse, and invertebrate models and show its utility for testing compounds that alter *SMN2 *splicing.

**Results:**

The human motoneuron disease SMA is caused by low levels, as opposed to a complete absence, of the survival motor neuron protein (SMN). To generate a true model of SMA in zebrafish, we have generated a transgenic zebrafish expressing the human *SMN2 *gene (*hSMN2*), which produces only a low amount of full-length SMN, and crossed this onto the *smn*^-/- ^background. We show that human *SMN2 *is spliced in zebrafish as it is in humans and makes low levels of SMN protein. Moreover, we show that an antisense oligonucleotide that enhances correct *hSMN2 *splicing increases full-length *hSMN *RNA in this model. When we placed this transgene on the *smn *mutant background it rescued the neuromuscular presynaptic SV2 defect that occurs in *smn *mutants and increased their survival.

**Conclusions:**

We have generated a transgenic fish carrying the human *hSMN2 *gene. This gene is spliced in fish as it is in humans and mice suggesting a conserved splicing mechanism in these vertebrates. Moreover, antisense targeting of an intronic splicing silencer site increased the amount of full length SMN generated from this transgene. Having this transgene on the *smn *mutant fish rescued the presynaptic defect and increased survival. This model of zebrafish SMA has all of the components of human SMA and can thus be used to understand motoneuron dysfunction in SMA, can be used as an vivo test for drugs or antisense approaches that increase full-length SMN, and can be developed for drug screening.

## Introduction

Identification of the survival motoneuron gene (SMN) as the genetic cause of the motoneuron disease spinal muscular atrophy (SMA) [1] was a major advance in the motoneuron disease field. It has enabled a way to model the disease in animals as a means to study disease biology and develop therapeutics. The genetics of SMA has been well characterized and supports that SMA arises from a deletion of the *SMN1 *gene and reliance for production of the SMN protein from the *SMN2 *gene [2-4]. The *SMN2 *gene, however, carries a number of nucleotide differences compared to *SMN1 *and one of these at position 6 in exon 7 results in a silent mutation that changes the splicing pattern of the gene [5,6]. The result is that the vast majority (~80-90%) of SMN from the *SMN2 *gene lacks exon 7 (SMNΔ7) [1]. This yields an unstable protein that cannot substitute for the full-length SMN protein [6-8].

Based on this information, an animal model of SMA needs both a deletion/dysfunction of the *SMN1 *gene and the presence of the *SMN2 *gene. An evolutionary analysis revealed that the *SMN1 *gene is duplicated in the chimpanzee genome, but only humans have the *SMN2 *gene [9]. Thus, it has been hypothesized that the human *SMN2 *gene (*hSMN2*) evolved from one of the *SMN1 *alleles. Since only humans have the *SMN2 *gene, the best way to generate an animal model of SMA is to add the *hSMN2 *as a transgene to an animal with a deleted/mutated *SMN1*. To date, this has been done in mice to generate a number of important models of SMA [2,3,7,10]. Models of SMA in drosophila, zebrafish, and Xenopus have relied on maternal Smn contributions [11,12] or transient Smn knockdown [13,14]. While these models are useful, they often have Smn levels that change during development and may not fully recapitulate the disease. To generate a complete model of SMA in zebrafish, we have generated transgenic zebrafish expressing the *hSMN2 *gene with its endogenous promoter. We then crossed this transgene into the previously characterized *smnY262stop*^-/+ ^line [12]. Here we show that *hSMN2 *is spliced in zebrafish consistent with what is seen in humans and mice [3]. In addition, we show that disrupting an intronic splicing silencer can increase the levels of full-length SMN from this transgene. The presence of the transgene results in a modest increase in SMN protein, a modest increase in survival compared to mutants lacking the transgene, and a delay in the presynaptic defect seen in *smn *mutant fish. Together these data show that we have generated a zebrafish model of SMA that has the genetics of human SMA.

## Results

### Characterization of transgenic *hSMN2 *zebrafish lines

**Figure 1:**
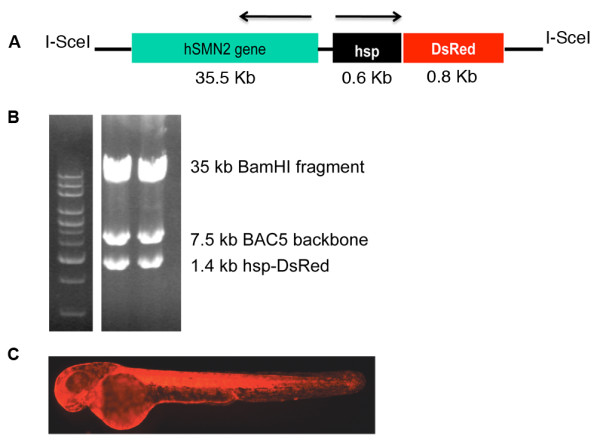
**Strategy for generating *Tg(hSMN2) *zebrafish**. **(A) **Diagrammatic representation of the *hSMN2:0.6hsp70:DsRed *DNA construct. Arrows indicate the direction of expression. **(B) **Digestion of the *hSMN2:0.6hsp70:DsRed *construct using BamHI on 0.5% agarose gel. Lane 1, high molecular weight DNA ladder (Invitrogen); lane 2-3, two examples of the *hSMN2:0.6hsp70:DsRed *construct. **(C) **Transgenic larvae (3 dpf) as revealed by heat shocking.

**Figure 2:**
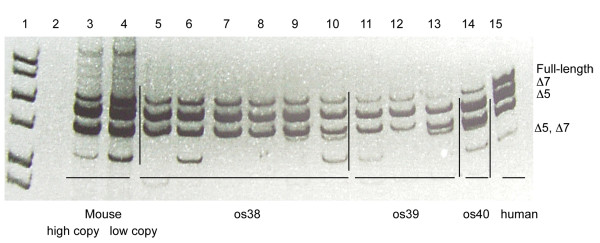
**Expression of *hSMN2 *RNA in transgenic zebrafish**. Representative polyacrylamide gel showing RT-PCR product from *hSMN2 *RNA in transgenic lines. Lane 1, 1 kb DNA ladder. Lane 2, negative control from a wild-type nontransgenic fish. Lanes 3 and 4, samples from spinal cords of low and high *SMN2 *copy transgenic mice [3], respectively. Lanes 5-10, single 3 dpf *Tg(hSMN2)os38 *larva. Lanes 11-13, single 3 dpf *Tg(hSMN2)os39 *larva. Lane 14, single 3 dpf *Tg(hSMN2)os40 *larva. Lane 15, human breast cancer cell line as a control.

**Figure 3:**
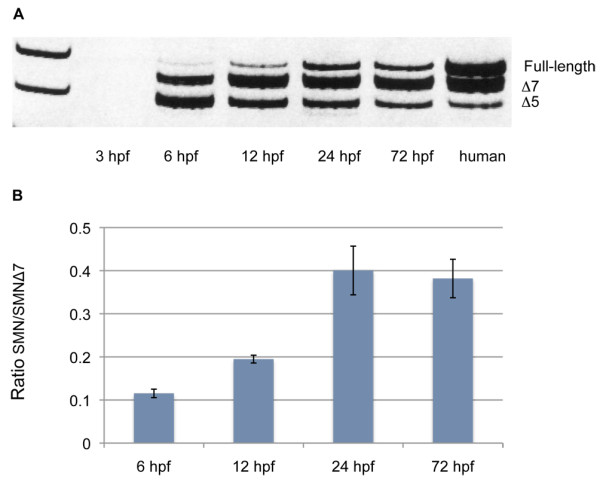
**Expression of *hSMN2 *RNA during embryonic development in transgenic zebrafish**. **(A)**Representative polyacrylamide gel showing RT-PCR product obtained from *Tg(hSMN2)os38 *RNA during early development. RNA from three embryos was prepared at 3, 6, 12, 24, and 72 hpf. Lane 1, 1 kb DNA ladder. Human, RNA from a human breast cancer cell line. **(B)** Quantification of the ratio of full-length *hSMN/hSMNΔ7 *from three separate experiments shown as mean ± sd.

To generate a complete model of SMA in zebrafish, we generated a transgenic zebrafish line expressing *hSMN2*. It had previously been shown that the entire human *hSMN2 *gene including its promoter was on a 35.5 kb *Bam*HI fragment in the genomic clone PAC 215P15 [3]. Recombineering [15] was used to clone the *Bam*HI fragment out of PAC 215P15 and tag the DNA with a 0.6 kb fragment of the zebrafish heat shock 70 promoter (0.6*hsp70*) [16] driving DsRed into pIndigoBac5. The *hsp70:DsRed *component was used for screening transgenics (Figure 1). The total construct therefore was *Tg*(*hSMN2;0.6hsp70:DsRed*), hereafter referred to as *Tg(hSMN2)*. This DNA construct was microinjected into early one-cell stage embryos. To determine which embryos received a high amount of DNA, injected embryos were heat shocked (37°C for 30 minutes) at 24 hours post fertilization (hpf). Only embryos expressing ubiquitous DsRed were grown for transgenic lines (~0.3-0.5%) thus increasing the chance of these fish having incorporated DNA into their germline. After injecting and screening, 38 F0s were grown to adulthood (~3 months) and then outcrossed to generate F1s. All F1s were heat shocked and those showing DsRed expression, indicative of the transgene, were grown to adulthood (Figure 1C). Approximately, 3% of the F0s gave rise to transgenic F1s. To examine *Tg*(*hSMN2*) expression, RNA obtained from 24 hpf transgenic embryos was used for RT PCR. *hSMN2 *was expressed in 10 transgenic zebrafish lines and 3 lines (os38, os39, os40) showed correct splicing of the *hSMN2 *gene (Figure 2). These splice products were characterized by both size and sequence. All three splice products, transcripts lacking exon 7 (Δ7), transcripts lacking exon 5 (Δ5), and transcripts lacking both (Δ5, Δ7) were present in all three lines. The splicing pattern was similar to the splicing pattern for endogenous *hSMN2 *from transgenic mice with low and high hSMN2 copy number [3] and from a human cell line (Figure 2A). The Δ7 and Δ5 forms were consistently present in these transgenic lines, but the Δ5, Δ7 form was more variable. The transgene copy number was estimated by quantitative PCR from DNA isolated from fin tissue. Hemizygous fish from *Tg(hSMN2)os38, Tg(hSMN2)os39, and Tg(hSMN2)os40 *had 30, 4, and 16 transgene copies respectively. RNA analysis from *Tg(hSMN2)os38 *revealed that full-length *hSMN *RNA is expressed as early as 6 hpf with an increase in the full-length *hSMN *between 6 and 24 hpf (Figure 3).

### Blocking an intronic splicing silencer site results in more full-length *hSMN *in *Tg(hSMN2) *fish

**Figure 4:**
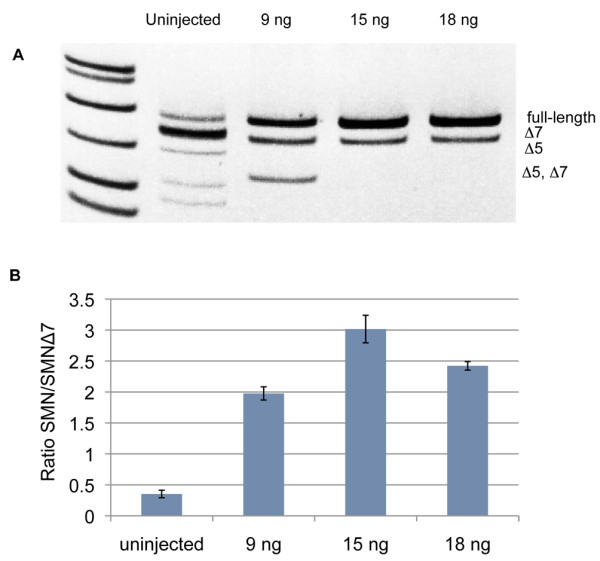
**Disrupting an intronic splice silencer site increases the amount of full-length *hSMN *RNA.(A)** Representative polyacrylamide gel showing RT-PCR product obtained from *hSMN *RNA exons 4-8. One-cell *Tg(hSMN2)os38 *embryos were injected with 9, 15 or 18 ng of *ISS-N1 *MO. RNA samples were prepared at 3 dpf for RT PCR. **(B) **Quantification of the ratio of full-length *hSMN/hSMNΔ7 *RNA from three separate experiments shown as mean ± sd.

An important therapeutic approach to SMA is to find methods to increase the amount of full-length SMN generated from the *SMN2 *gene. One promising approach that has recently been tested in vivo is to use antisense oligonucleotides against an intronic splicing silencer (ISS) site in intron 7 [17] to increase exon 7 inclusion and thus generate more full-length SMN from the *SMN2 *gene [18]. To test whether the *Tg(hSMN2) *zebrafish could be used as a tool to test this technology, we injected an antisense *ISS-NI *morpholino (MO) into 1-cell stage *Tg(hSMN2)os38 *embryos. At 3 days post fertilization (dpf) RNA was harvested and the human *SMN *full-length and *SMN*Δ*7 *levels generated from the transgene were analyzed by RT-PCR and quantified by measuring band intensity (Figure 4). Uninjected embryos had a full-length *SMN/SMN*Δ*7 *ratio of 0.36 ± 0.05 (mean ± sd). When we injected 9 ng of *ISS-NI *MO, the ratio increased ~5-fold to 1.9 ± 0.13 and the ratio increased ~10-fold (3.45 ± 0.54) with the 15 ng dose. When we injected the highest dose, 18 ng, the ratio increased ~7-fold (2.9 ± 0.34) however the embryos showed some developmental abnormalities suggesting that this dose was too high. This increase was transient, however, and was no longer present at 7 dpf (data not shown). This is not surprising since morpholino perdurance is variable and most reports indicate early phenotypes within 3-5 dpf [19]; although each morpholino is unique and some can affect protein levels into the second week of development [13]. These data show that *Tg(hSMN2) *zebrafish respond to splicing factors similarly to mice carrying the human transgene and can serve as a fast and easy in vivo system to test approaches to increase full-length *SMN *from the *hSMN2 *gene.

**Figure 5:**
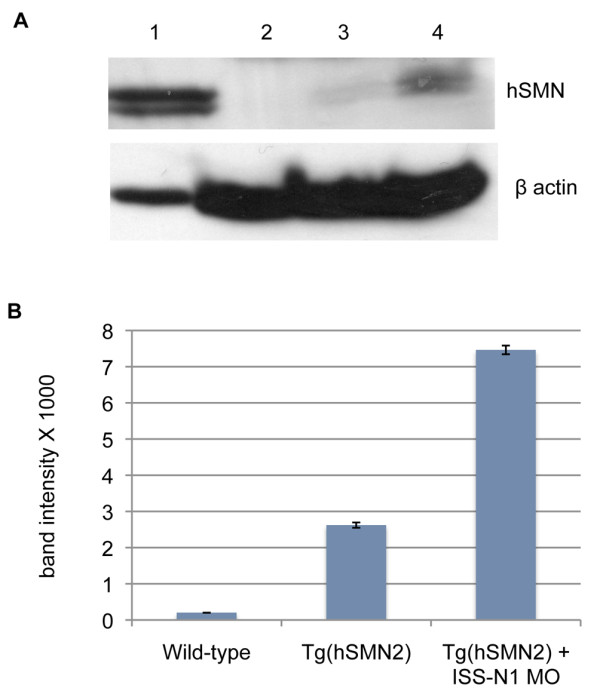
**hSMN protein in *Tg(hSMN2) *larvae**. **(A)** Representative western blot. Lane 1, 10 day old *Smn*^*-/-*^*;SMN2*^*+/+*^*;delta7*^*+/+ *^mouse brain (10 μg); Lane 2-4, wt, *Tg(hSMN2)os38*, and *Tg(hSMN2)os38 *+ 15 ng *ISS-N1 *MO zebrafish larvae collected at 3 dpf (150 μg of protein per lane). The blot was probed with human SMN [18] and β-actin monoclonal antibodies. **(B)** Quantification of hSMN band intensity from two experiments shown as mean ± sd.

We next asked whether this change in splicing increased the amount of full-length protein generated from the transgene. For this we used a human specific SMN antibody [18]. To determine whether hSMN was being made from the transgene, we first analyzed 3 dpf *Tg(hSMN2)os38 *and found that hSMN was present at low levels (Figure 5). Next, we analyzed protein from 3 dpf *Tg(hSMN2)os38 *larvae injected with *ISS-N1 *MO at the 1-2 cell stage. Here we found an ~ 3-fold increase in hSMN protein. These data show that hSMN is made in *Tg(hSMN2) *and disrupting a splicing motif can increase the levels.

### *Tg(hSMN2) *extends survival when crossed into *smn *mutant fish

**Figure 6:**
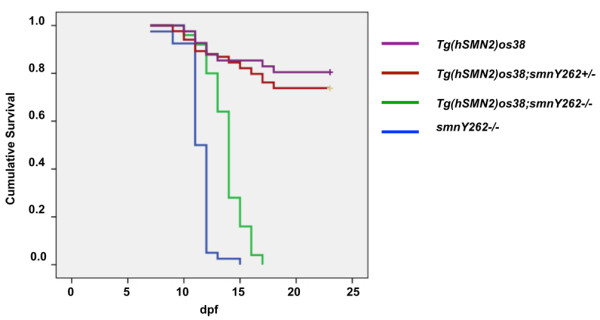
**hSMN2 extends the survival of *smnY*262*stop*^*-/- *^fish**. Survival was followed up to 23 dpf and a Kaplan-Meier plot was generated. *Tg(hSMN2)os38*, purple line, *n *= 143; *Tg(hSMN2)os38; smnY262stop*^*+/-*^, red line, n = 62; *Tg(hSMN2)os38; smnY262stop*^*-/-*^, green line, *n *= 25; *smnY262stop*^*-/-*^, blue line, *n *= 40. The average survival was 12 dpf (range = 7-15 dpf) for *smnY262stop*^*-/- *^larvae and 14.7 dpf (range = 10-17 dpf) for *Tg(hSMN2)os38; smnY262stop*^*-/- *^(*p *< 0.0001).

Small changes in full-length SMN can alter disease phenotypes in patients and in animal models [20,21]. We therefore asked whether the presence of the *hSMN2 *transgene and the presence of a small amount of hSMN protein could extend survival of *smn *mutants, For these experiments we utilized zebrafish *smn *stop mutation, *smnY262stop*. We had previously shown that *smnY262stop*^-/- ^die at 12 dpf (range: 9-16 dpf) [12]. To determine whether the modest increase in hSMN extended survival of *smnY262stop*^-/- ^mutants, we analyzed survival of *Tg(hSMN2)os38;smnY262stop*^-/- ^fish. To generate these lines, *Tg(hSMN2)os38;smnY262stop*^-/+ ^were outcrossed to *smnY262stop*^+/- ^and embryos were heat shocked at 1 dpf and screened at 2 dpf for DsRed fluorescence indicative of the transgene. DsRed positive embryos and DsRed negative embryos were identified and monitored over 23 days. Any larvae that died were kept and on day 23, all fish were genotyped to identify the *smnY262stop*^*-/- *^larvae from both the positive and negative transgene populations. We found that the *hSMN2 *transgene slightly extended the survival of *smnY262stop*^*-/- *^from an average of 12 (9-16) to14.7 (10-17) dpf (*p *< 0.0001) (Figure 6). This increase in survival is consistent with the slight increase in full-length hSMN from the *Tg(hSMN2)os38 *gene seen by Western blot (Figure 5). We did not, however, see any difference in the survival of *Tg(hSMN2)os38*; *smnY262stop*^-/- ^injected with the *ISS-N1 *MO (n = 120 larvae) (data not shown). This is consistent with our finding that the increase in full-length hSMN in *Tg(hSMN2)os38 ISS-NI *MO injected embryos was transient and gone by 7 dpf (data not shown). These data suggest that this transient increase in hSMN was not enough to affect later events such as survival.

### hSMN2 rescues the presynaptic NMJ defect

**Figure 7:**
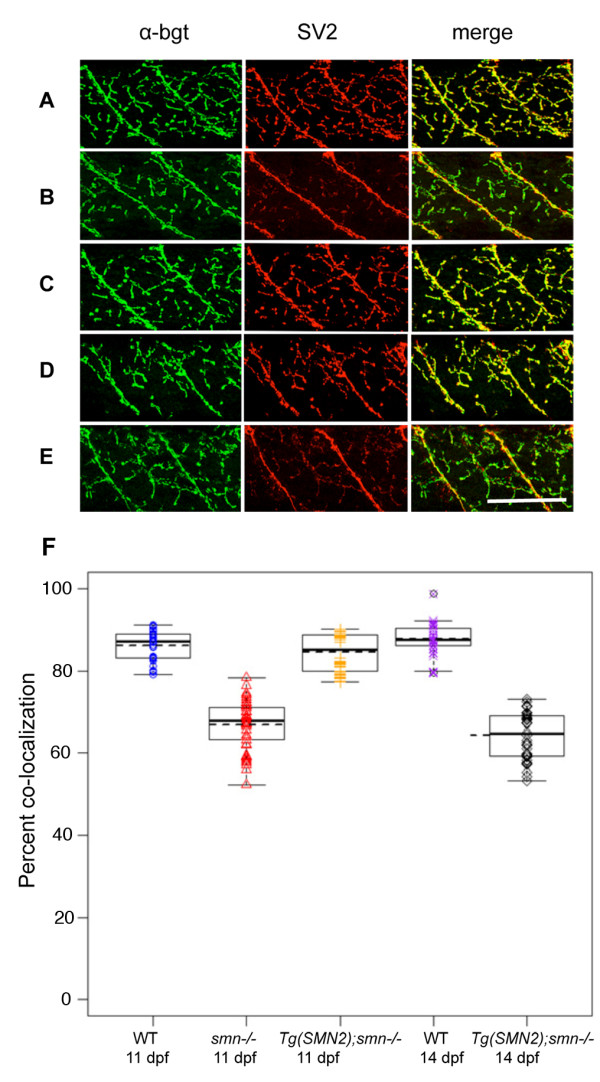
***Tg(hSMN2*) rescues the NMJ defect in *smn *mutants**. Immunolabeling of presynaptic regions (SV2, red) and postsynaptic regions (α-bgt, green) of **(A) **11 dpf WT larvae (n = 10); **(B) **11 dpf *smnY262stop*^*-/- *^larvae (*n *= 13); **(C) **11 dpf *Tg(hSMN2)os38;smnY262stop*^*-/- *^larvae (*n *= 12); **(D) **14 dpf WT larvae (n = 8) **(E) **14 dpf *Tg(hSMN2)os38; smnY262stop*^*-/- *^larvae (*n *= 12); Merge is the overlay. (**F**) The coefficients of co-localization were plotted and the means of each group were calculated. *Tg(hSMN2)os38; smnY262stop*^*-/- *^were significantly different compared to the *smnY262stop*^*-/- *^at 11 dpf (*p *< 0.0001). At 14 dpf, there was a significant difference between the *Tg(hSMN2)os38;smnY262stop*^*-/- *^and wild types. Scale bar, 80 μm.

We have shown that presynaptic terminals are present in zebrafish *smn *mutants, but there is a decrease in SV2 protein at the NMJ [12]. Our hypothesis is that SV2 loss at the NMJ is an indication that the synapse is becoming compromised. To ask whether SV2 loss is observed in *Tg(hSMN2)os38*;*smn*^*-/- *^larvae, we analyzed SV2 expression (Figure 7). We crossed *Tg(hSMN2);smnY262 stop*^*+/- *^to *smnY262stop*^*+/- *^and larvae carrying the transgene were identified by heat shock and raised to 11 dpf. At this time they were genotyped and immunolabeled with SV2 and α-bgt. In *Tg(hSMN2)os38;smnY262*^*-/- *^at 11 dpf, SV2 and α-bgt were co-localized and no NMJ defects were observed compared to *smnY262stop*^*-/- *^larvae that had an overall reduction in SV2 as compared to wild-types (Figure 7A-B, F). Introduction of *hSMN2*, however, rescued the SV2 reduction seen in homozygous *smn *mutants (Figure 7C, F). We repeated the immunolabeling at 14 dpf and observed SV2 defects similar to those seen in the homozygous *smn *mutants (Figure 7E, F). To quantify the NMJ defects, we plotted the co-localization efficiency of the pre- and postsynaptic regions. These data are consistent with our previous observation [12] indicating that when SMN levels are low, presynaptic NMJ changes occur.

## Discussion

Here we characterize the generation and characterization of a zebrafish having both genetic components of SMA; that is, a mutation in the endogenous *smn *gene and the presence of the human *SMN2 *gene. We show that *hSMN2 *is spliced correctly in zebrafish and that it contributes a small amount of full-length hSMN. This increase in SMN protein statistically improved survival, albeit by only a few days, and rescued the presynaptic NMJ defect for that same number of days. We also show using the *Tg(hSMN2) *line that we can modulate the amount of full-length *hSMN *RNA by disrupting an intronic splicing silencer site with an antisense MO. Thus, this model has utility both as a vertebrate model of SMA and as a way to test approaches to increase full-length SMN from the *SMN2 *gene in vivo.

The finding that the presence of the *Tg(hSMN2)os38 *transgene only increased survival by ~2 days is due to the fact that it only increased full-length protein by a small amount. Because we have multiple lines that all express at low levels, this is not likely due to integration sites, but more likely caused by the *hSMN *promoter not being very efficient in zebrafish or human RNA not being translated efficiently in zebrafish. To further increase the amount of full-length SMN in this model, we can increase the copies of the *hSMN2 *transgene by crossing in additional copies of the *hSMN2 *gene. However, it does appear that the human *SMN2 *promoter is not very robust in zebrafish as our highest expressing line (os38) has 30 copies of the transgene and only a slight increase in SMN protein. It is also possible that some of these copies are silenced [22]. Data from human patients and mouse models of SMA show, however, that even slight increases in SMN increase survival and decrease disease severity. This is supported by data presented here showing that a slight increase in protein can cause a corresponding increase in survival and a delay in presynaptic defects.

In previous experiments, we showed that driving *hSMN *cDNA only in motoneurons (using the zebrafish *hb9/mnx1 *promoter) rescued the presynaptic NMJ defect, but not survival [12]. It is not surprising that survival was not rescued since SMN is needed in all cells, even at low levels, or the organism will die [23]. In the model presented here, SMN present at low levels in all cells extended survival and rescued the SV2 presynaptic defect during that extension. However, before the fish died, their SV2 decreased much as it did in mutants lacking the transgene [12] (Figure 7). These data support our earlier conclusion that low levels of SMN lead to changes at the NMJ presynaptic terminal [12]. This is also consistent with mouse models of SMA that show poor presynaptic terminal differentiation [24], decreased density of synaptic vesicles [25], and evidence of unoccupied synapses [26]. Drosophila models also show evidence of NMJ defects [11]. Thus, across species, low levels of SMN result in NMJ defects.

These data show that we have generated a complete genetic model of SMA in zebrafish. This is only the second model organism where this has been accomplished. Having this model in zebrafish complements the mouse models and also provides the ability to perform different types of experiments. For example, it is very standard in zebrafish to generate genetics mosaics to address issues of cell autonomy [27,28]. Since large numbers of embryos can be collected, this system is also amenable to drug screening [29]. Moreover, as we show here this model can be used to quickly and easily test compounds to determine their affect on *hSMN2 *splicing in vivo.

## Methods

### Zebrafish maintenance

Adult zebrafish and embryos were maintained by standard protocols [30]. All fish were maintained at temperatures between 27 and 29°C. Zebrafish used for making transgenics were on the *AB/LF background. Characterization of the *smnY262stop *mutant has been previously described [12].

### Cloning and recombineering

Two 540 bp fragments (arms) from human PAC215p15 (AC004999) were amplified using PCR for recombineering [15]. These arms were complementary to sequences outside of the 35.5 kb fragment that contained *SMN2*. The 5' arm was amplified by PCR using forward primer: 5'AGTGAGCTCAAGCATTCTTATACACCACCC; reverse primer: 5'GGACACGCGTTGTCAAAGATCAGATAGTTG and digested with Sac I and Mlu I. The 3' arm was amplified using PCR forward primer: 5'ACTACGCGTGATCCTGTGGCTTCAATGTCAT; reverse primer: 5' CAGCAAGCTTCAGGATATGATCTCCATACAG and digested with Mlu I and Hind III. These two digested PCR products were then triple cloned into SacI and Hind III sites of the pBluescriptSK (pBSK) vector which contained two Sce I sites (gift from Dr. Bruce Appel) and referred to as the pBSK arm vector. The EGFP from pEGFP-1 backbone (Clontech) was removed at Sal I and Not I sites and replaced by DsRed. The last 0.6 kb of the zebrafish *hsp70 *promoter generated from the EcoR1 site inside the full promoter [16] was amplified with DsRed using primer forward 5'ATATAAGCTTACTGGAGGCTTCCAGAACAG and reverse 5'GCCTCGAGCTTAAGATACATTGATGAGTTTG. The PCR product was digested with Hind III and Xho I and cloned into the pBluescript arm vector.

The entire fragment containing two Sce I sites, two arms and 0.6 kb of the Hsp70-DsRed was amplified using PCR with forward primer 5'TAAGGATCCCACGGAAACAGCTATGACC and reverse primer 5'ATAGGATCCCACGACGTTGTAAAACGACG. The entire PCR product was digested with Bam HI and cloned into pIndigoBac5 (Epicentre). This DNA plasmid was digested with Mlu I and 5 ng of digested DNA was used for transformation. One colony of PAC215p15 in SW102 cells was grown overnight at 32°C in 3 ml culture containing kanamycine. The culture was diluted to 1% in 15 ml and grown at 32°C until it reached OD = 0.6 and then shaken at 42°C for 15 minutes. The culture was chilled on ice for 10 minutes and washed twice in ice-cold water. The cell pellet was eluted in 100 μl of water and used for electro transformation with the above digested plasmid and spread onto cloramphenicol agar plates and grown overnight at 32°C. Colonies were cultured at 32°C and plasmids were screened for the 35.5 kb fragment by digesting with Bam HI.

### Generation of transgenics

DNA injections were performed as described [31]. Plasmid DNA was prepared (Qiagen Plasmid Midi kit) and diluted to 200 ng/μl in I-SceI buffer containing 10 mM Tris-HCl, 1 mM dithiothreitol, 10 mM MgCl_2_, pH 8.8, 5 Units of SceI enzyme (New England Biolab) and 0.1% phenol red. Sample was prepared fresh before each injection. DNA (200 ng/μl) was injected into embryos at the early one-cell stage to 10% of the volume of the cell (~ 1 nl). Injected embryos were transferred into fish water containing penicillin/streptomycin (Invitrogen) 1/100. Injected fish (F0s) were heat shocked at 1 dpf at 37°C for 30 minutes and screened at 2 dpf. To increase the likelihood that the transgene would go germline, only F0s expressing DsRed in close to 100% of cells were kept and grown to adulthood. Once they reached adulthood, F0s were outcrossed to wild-type fish and the resulting F1s were heat shocked and screened for DsRed fluorescence. F1s were grown to adulthood and outcrossed to generate transgenic lines. F1s from the same F0 were kept as separate lines since they could arise from transgene insertion into different germ cells. Transgenic lines were designated as: Tg(*hSMN2;0.6hsp70:DsRed) *followed by the lab designation (os for Ohio State) and a line number.

### RNA extraction, RT PCR, and sequencing of *SMN2 *transcripts

Total RNA from zebrafish embryos and larvae was isolated using Trizol reagent (Invitrogen) following the manufacturer's protocol. RT-PCR was performed on 10 ng of total RNA using a Quigen one-step RT-PCR kit. RNA from the *hSMN2 *gene was amplified by human specific primers in exon 4 and 8 as in Le et al. [7]: 5'-GTGAGAACTCCAGGTCTCCTGG-3' and 5'-CTACAACACCCTTCTCACAG-3'. Human breast cancer cell line MCF10CA1a was used as a control (gift from the Hai lab). PCR products were run on 8% polyacrylamide gel. Images were captured by Gel Doc 2000 (Bio Rad). The images were scanned and the intensity of the full-length *hSMN *and *hSMN7 *bands were determined by Photoshop Element 5.0. The ratios of intensity of full-length *hSMN *and *hSMNΔ7 *were reported.

For sequencing *hSMN2 *transcripts, RT-PCR was performed on total RNA extracted from ten 3 dpf *Tg(hSMN2)os38 *larvae using human specific primers in exon 4 and 8 [7]. The PCR product was run on a 1% agarose gel and the four bands excised and purified using Qiagen gel extraction kit. Purified PCR products were then cloned into PCR8/GW/Topo vector (Invitrogen) using PCR8/GW/Topo TA cloning kit (Invitrogen). DNA plasmids were sequenced using primer M13F with sequence GTAAAACGACGGCCAG. The sequencing results were blasted to NCBI Reference Sequence: NM_000344.3.

### Quantitative PCR

DNA was extracted from adult fish fins using DNeasy Tissue Kit (Qiagen). Quantitative PCR (qPCR) was performed as described in Ramesh et al, 2010 [32]. The h*SMN2 *transgene was detected with primers to amplify intron2: SMN2F2 5'-GCGATAGAGTGAGACTCCATC and SMN2R1 5'-GACATAGAGGTCTGATCTTTAGCT. Fish β*-actin *F primers: 5'-CATGAGACCACCTTCAACTCC and fish β*-actin *R primer: 5'-TGAAATCACTGCAAGCAAACTG were used to amplify the endogenous β*-actin *gene.

DNA from low and high copy *SMN2 *transgenic mice [3] and DNA from human breast cancer cell line MCF10CA1a was used as a control. The mouse β*-actin *gene was amplified using mouse β-actin F 5'-GTATGGAATCCTGTGGCATCC and mouse β-actin R 5'-ATACAAGATGGTGAATGGTGAG primers. The qPCR was performed on the iCycler (Bio-rad) with IqSYBR Green Supermix (BioRad) containing 5 ng of genomic DNA and 10 pmol of each primer. The data was analyzed as described [32].

### Antisense oligonucleotides injection

A morpholino directed against an intron splice silencer (ISS-N1) site with sequence ATTCACTTTCATAATGCTGG [18] was purchased from Gene Tools. The stock was diluted to 2 mM in dH2O. One-cell stage *Tg(hSMN2)os38 *embryos were injected with 9, 15 and 18 ng of the *ISS-N1 *MO using an MPPI-2 Pressure Injector (Applied Scientific Instrumentation). Injection of these three doses was repeated three times. At 3 dpf total RNA from injected and uninjected zebrafish embryos was isolated using Trizol reagent (Invitrogen) following the manufacturer's protocol. RT-PCR was performed twice for each sample for a total 6 RT-PCR reactions per treatment. The products were run on 8% polyacrylamide gel and images were captured by Gel Doc 2000 (Bio Rad). The images were scanned and the intensity of the full-length *hSMN *and *hSMNΔ7 *bands were determined by Photoshop Element 5.0. The ratios of intensity of full-length *hSMN *and *hSMNΔ7 *were reported.

### Survival assay and genotyping

The survival assay was performed on progeny from crosses between heterozygote *Tg(hSMN2)os38 *and *smn262*^+/- ^fish. Progeny from these crosses were heat shocked at 1 dpf and screened at 2 dpf. *Tg(hSMN2)os38 *were fluorescent and kept for analysis. Those without a DsRed signal were kept as the control group. Both groups were raised in the same nursery environment. The dead larvae were collected twice a day and frozen. At 23 dpf, the remaining larvae were sacrificed and all larvae, including those that died earlier, were then genotyped as described [12]. For each fish time of death, survival status, and classification were put into SPSS (SPSS version 15; SPSS, Chicago, IL, USA). Kaplan-Meier survival tests were run to generate the survival curve and *p*-values were calculated by the log-rank test.

### Western blotting

Six zebrafish embryos (3 dpf) of wild-type, *Tg(hSMN2)os38 *and *Tg(hSMN2)os38 *injected with 15 ng *ISS-N1 *MO were dissolved in 10 μl of blending buffer (62.6 mM Tris pH 6.8, 5 mM EDTA and 10% SDS) and boiled for 10 minutes. The samples were then diluted with an equal volume of loading buffer (100 mM Tris pH 6.8, 4% SDS, 0.2% Bromophenol Blue, 20% glycerol and 200 mM dithiothreitol), boiled for 2 minutes. The whole amount of each sample from six embryos (~150 μg) and ~10 μg of brain protein from 10 day *Smn*^*-/-*^*;SMN2*^*+/+*^*;delta7*^*+/+ *^mice [33] were resolved on a 12.5% polyacrylamide gel. The gel was electrotransfered to the Protran BA 83 Nitrocellulose membrane (Whatman). Membranes were probed with mouse monoclonal antibodies: human specific SMN-KH monoclonal antibody [18] (1/20) or anti-actin (Santa Cruz) (1/5000). Signal was detected with horseradish peroxidase-conjugated goat anti-mouse antibody (1/5000) (Jackson ImmunoResearch Laboratories, Inc), ECL reagents and Amersham Hyperfilm ECL (Amersham Bioscience). The images were scanned from two separate experiments and the intensity of the bands determined by Photoshop Element 5.0 and reported as mean ± sd.

### Immunofluorescence staining and confocal microscopy

Zebrafish larvae were anesthetized with tricain (Sigma, A-5040). The head of each larva was removed for genotyping as described [12]. The body was fixed in 4% paraformaldehyde in PBS and 1% DMSO overnight at 4°C. Larvae were then washed in 1XPBS for 10 minutes, distilled H_2_O for 10 minutes followed by a 15 minute incubation at room temperature with -20°C Acetone. Samples were then washed with distilled H_2_O for 20 minutes. Postsynaptic regions were immunostained for 1 hour with α-bgt conjugated to Alexa Fluor 488 (Invitrogen) diluted 1/100 in PBDT buffer (1XPBS, 1% DMSO, 1% BSA, 0.5% TritonX-100) and 2.5% normal goat serum as in [12]. Samples were washed for 10 minutes 5X in PBST (0.5% TritonX-100 in 1XPBS). Samples were then incubated overnight at 4°C with presynaptic antibody SV2 diluted 1/100 in PBDT buffer and 2.5% normal goat serum. Samples were washed 5 × 10 minutes with PBST at room temperature and incubated overnight at 4°C with Alexa Fluor 633 goat-anti mouse IgG (Invitrogen) diluted 1/400 in PBDT and 2.5% normal goat serum. Samples were washed for 5 × 10 minutes in PBST, mounted on a slide with vectashield (Vector Labs, Burlingame, CA, USA) and images were captured with the Leica TCS SL scanning confocal microscope system. Neuromuscular junction (NMJ) analysis was performed as described [12]. Changes in the co-localization coefficients and log ratios of pre- and postsynaptic only regions determined by NIH Image J were analyzed using a one-tailed Mann-Whitney U test (R 2.6.0; GNU project).

## List of abbreviations

SMA: spinal muscular atrophy; SMN: survival motor neuron; α-bgt: α-bungerotoxin; hpf: hours post fertilization; dpf: days post fertilization.

## Competing interests

The authors declare that they have no competing interests.

## Authors' contributions

HL performed all of the experiments and performed data analysis, AHMB helped with the conceptual design of the transgene and supplied reagents and the *ISS-NI *MO, CEB was involved in the conceptual design of the experiments, data analysis, and data interpretation. CEB and HL wrote the manuscript. All authors have read and approved the final manuscript.
